# Associations of Serum Cystatin C, DNAm Cystatin C, Renal Function, and Mortality in U.S. Adults

**DOI:** 10.3390/life15010013

**Published:** 2024-12-27

**Authors:** Yu-Wei Fang, Wei-Chung Huang, Chikang Wang, Chien-Yu Lin

**Affiliations:** 1 Division of Nephrology, Department of Internal Medicine, Shin-Kong Wu Ho-Su Memorial Hospital, Taipei 111, Taiwan; m005916@ms.skh.org.tw; 2 School of Medicine, College of Medicine, Fu Jen Catholic University, New Taipei City 242, Taiwan; 3 Department of Obstetrics and Gynecology, En Chu Kong Hospital, New Taipei City 237, Taiwan; 01603@km.eck.org.tw; 4 Department of Environmental Engineering and Health, Yuanpei University of Medical Technology, Hsinchu 300, Taiwan; ckwang@mail.ypu.edu.tw; 5 Department of Internal Medicine, En Chu Kong Hospital, New Taipei City 237, Taiwan

**Keywords:** cystatin C, epigenetic biomarker, DNAmCystatinC, chronic renal failure, eGFRcr-cys, mortality

## Abstract

Serum cystatin C is a well-established marker of renal function and a valuable predictor of health risks and mortality. DNA methylation-predicted cystatin C (DNAmCystatinC), an advanced epigenetic biomarker, serves as a proxy for serum cystatin C levels. However, the relationships between serum cystatin C, DNAmCystatinC, renal function, and mortality outcomes have not been previously examined. This study aimed to examine the associations between serum cystatin C, DNAmCystatinC, renal function, and their joint and independent relationships with mortality in U.S. adults. We analyzed data from 1642 participants aged 50 and older from the National Health and Nutrition Examination Survey (NHANES) 1999–2002, linked to mortality information from the National Center for Health Statistics (NCHS), with follow-up through 2019. Our analysis demonstrated a positive association between ln-DNAmCystatinC and ln-serum cystatin C (Adjusted β (SE) = 0.773 (0.267), *p* = 0.007), while ln-DNAmCystatinC was negatively correlated with ln-Estimated glomerular filtration rate, calculated using both creatinine and cystatin C (eGFRcr-cys) (Adjusted β (SE) = −1.123 (0.449), *p* = 0.018). In a weighted Cox regression model, a one-unit increase in ln-serum cystatin C was linked to an increased hazard ratio (HR) of 2.87 (95% CI: 1.938–4.26, *p* < 0.001) for all-cause mortality and 3.04 (95% CI: 1.34–6.88, *p* = 0.010) for cardiovascular mortality. Additionally, a one-unit increase in ln-DNAmCystatinC was associated with an HR of 135.86 (95% CI: 5.51–3349.69, *p* = 0.004) for all-cause mortality. This association was particularly pronounced in participants without chronic kidney disease (CKD), with a *p*-value for the interaction between DNAmCystatinC and CKD on all-cause mortality of 0.002. Furthermore, individuals with serum cystatin C and DNAmCystatinC levels above the 50th percentile showed the highest all-cause mortality risk when compared to other subgroups. In conclusion, our findings demonstrate that DNAmCystatinC is a stronger predictor of all-cause mortality than serum cystatin C, with potential additive effects when both biomarkers are considered together. These results suggest their utility as valuable clinical indicators for risk stratification and early intervention. Future research should validate these findings and further explore the clinical and public health implications of epigenetic biomarkers.

## 1. Introduction

Chronic kidney disease (CKD) is a major global health issue, ranking as the seventh-leading cause of death from non-communicable diseases [1]. Its prevalence has increased from 11% in the 1990s to over 15% in recent years, with a 41.5% rise in mortality between 1990 and 2017 [2]. CKD elevates the risk of cardiovascular disease (CVD) and all-cause mortality, even after accounting for traditional risk factors [3]. Early detection of CKD through surveillance programs can slow disease progression and improve patient outcomes [4]. In clinical practice, the estimated glomerular filtration rate (eGFR) is essential for assessing kidney function, typically using biomarkers like creatinine and cystatin C [5]. While creatinine-based equations are widely used, they have limitations due to factors like muscle mass [6]. Cystatin C is a low-molecular-weight protein produced by all nucleated cells and freely filtered by the glomeruli, making it a reliable indicator of eGFR [7]. eGFR calculated using both creatinine and cystatin C (eGFRcr-cys) is generally more accurate and reliable than using either biomarker individually [8]. Beyond assessing renal function, cystatin C regulates tissue remodeling, immune responses, and inflammation by inhibiting cathepsins involved in protein degradation. Its broad biological roles make it a valuable biomarker not only for renal function but also for cardiovascular, neurodegenerative, and inflammatory diseases [9,10]. Serum cystatin C levels have proven to be a promising predictor of CVD in individuals with normal or mildly impaired kidney function [11,12].

Epigenetic mechanisms—such as DNA methylation, histone modifications, and RNA-associated editing—regulate gene expression without changing the DNA sequence [13]. These processes play vital roles in human biology, health, and evolution, with environmental factors influencing them and potentially causing transgenerational effects that impact long-term health [14]. Recent studies highlight genome-wide DNA methylation profiles as valuable tools for cancer detection, diagnosis, and prognosis, as well as for assessing aging and age-related conditions [15]. Among these tools is GrimAge, an advanced epigenetic clock that estimates biological age and mortality risk by analyzing DNA methylation patterns and incorporating markers like inflammation and smoking history [16]. These markers have also been linked to CKD and reduced eGFR, indicating a potential connection between epigenetic aging processes and kidney function [17]. Among these biomarkers, DNA methylation-predicted cystatin C (DNAmCystatinC) has gained attention as a proxy for serum cystatin C levels. Unlike serum cystatin C, which directly measures protein levels in the bloodstream, DNAmCystatinC is derived from DNA methylation patterns. Integrated into advanced biological aging models like GrimAge and GrimAge2, DNAmCystatinC enhances predictive accuracy for mortality and aging-related outcomes [18]. Elevated levels of DNAmCystatinC have been associated with major depressive disorder, highlighting its relevance across multiple health domains [18].

Despite these advancements, the clinical utility of DNAmCystatinC remains underexplored, particularly concerning its role in predicting mortality outcomes. While broader epigenetic markers like GrimAge have shown associations with kidney function and mortality [16], specific insights into DNAmCystatinC remain limited. Crucially, no studies have directly compared the predictive power of serum cystatin C and DNAmCystatinC for mortality, nor have they assessed whether the combined use of these two biomarkers offers additive predictive value for mortality risk. To address this gap, we analyzed data from the 1999–2002 National Health and Nutrition Examination Survey (NHANES), linked to mortality outcomes tracked by the National Center for Health Statistics (NCHS) through 2019. This dataset provides a unique opportunity to explore the relationships between serum cystatin C, DNAmCystatinC, eGFRcr-cys, and mortality outcomes within a representative U.S. adult population. Our study aims to offer the first epidemiological insights into these associations, with a focus on their clinical implications for risk stratification and early intervention.

## 2. Materials and Methods

### 2.1. Study Population

**Figure 1:**
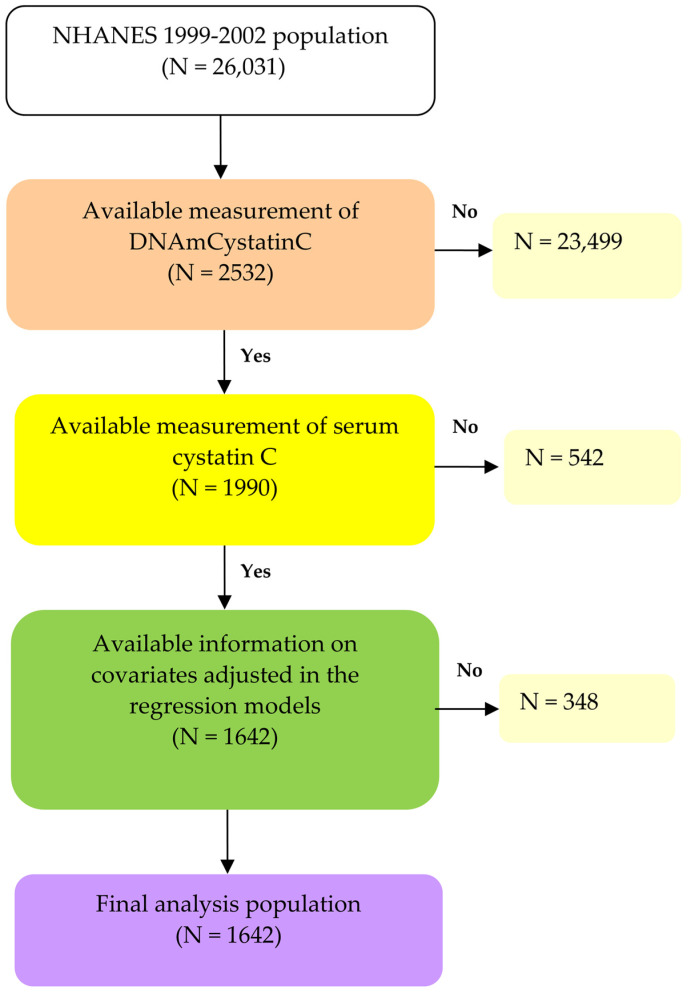
Flow chart algorithm.

The National Health and Nutrition Examination Survey (NHANES) is conducted every two years to provide a comprehensive, nationally representative snapshot of the non-institutionalized civilian population in the United States. Utilizing a complex, multistage probability sampling design, NHANES ensures that its results accurately reflect the broader U.S. population. The dataset used in this study is publicly accessible and de-identified, adhering to strict privacy and ethical guidelines established by the Centers for Disease Control and Prevention. Researchers worldwide can access and analyze NHANES data under the established data usage agreement. Detailed descriptions of the survey’s protocols and consent procedures can be found on the NHANES website [19]. This study utilized data from the 1999–2002 NHANES cycle, which initially included 26,031 participants. Of these, DNAmCystatinC measurements were available for 2532 individuals, and serum cystatin C levels were recorded for 1990 of them. After applying eligibility criteria based on covariates, 1642 participants were included in the final multiple regression analysis. The selection flow is outlined in Figure 1.

### 2.2. Measurement of DNAmCystatinC

DNAmCystatinC was retrospectively derived from DNA methylation data obtained from archived biospecimens. The study included adults aged 50 and older from the NHANES 1999–2002 surveys who had provided stored blood samples for DNA analysis. The sample consisted of a random selection of approximately half of the eligible non-Hispanic white participants, as well as all eligible other racial/ethnic groups. For DNAm analysis, 500 ng of bisulfite-treated DNA was processed using the Illumina Infinium Methylation EPIC BeadChip (Illumina, San Diego, CA, USA). The DNA samples underwent hybridization, amplification, and imaging via the Illumina iScan system (Illumina, San Diego, CA, USA). Data preprocessing was conducted in RStudio (version 2023.03.01) with R (version 4.3.1), which involved converting Illumina data files into methylation signals. DNAmCystatinC values were calculated, normalized, and filtered to ensure probe accuracy and performance, while technical variations were corrected using ComBat (Johns Hopkins University, Baltimore, MD, USA). Full descriptions of the analytical procedures can be found on the NHANES website [20].

### 2.3. Measurement of Serum Cystatin C

All participants aged 60 and older with available samples, along with a randomly selected 25% sample of those aged 12–59, were included. This current analysis specifically focused on individuals aged 50 and above. The cystatin C levels were measured using the Dade Behring N Latex Cystatin C assay, an automated particle-enhanced nephelometric assay performed on the Dade Behring Nephelometer II. The assay demonstrated an intra-assay coefficient of variation between 2.0% and 3.0%, and an inter-assay coefficient of variation ranging from 3.2% to 4.4%. The detectable range of the assay was 0.23–7.25 mg/dL. For results falling below the LOD, a value equal to the LOD divided by the square root of 2 was used. Full details of the assay procedures are available on the NHANES website [21].

### 2.4. Covariates

Data on sociodemographic variables, including age, gender, and ethnicity, were sourced from the NHANES database. Smoking status was classified into three categories: current smokers, individuals exposed to environmental tobacco smoke (ETS), and non-smokers, based on responses from a smoking questionnaire and serum cotinine measurements [22]. Alcohol use was evaluated through a questionnaire asking whether participants had consumed at least 12 alcoholic drinks in the past year. Body mass index (BMI) was calculated by dividing weight (kg) by height (m^2^). Hypertension was determined either by the self-reported use of blood pressure medications or by an average blood pressure reading of ≥140/90 mmHg. Diabetes was defined as a fasting glucose level of ≥126 mg/dL, a glycated hemoglobin level of ≥6.5%, or the self-reported use of antidiabetic drugs. Hypercholesterolemia was diagnosed based on a fasting low-density lipoprotein cholesterol level of ≥130 mg/dL or current treatment for high cholesterol. In this study, eGFRcr-cys was measured using the equation established by the National Kidney Foundation) [23]. CKD was identified by eGFRcr-cys less than 60 mL/min/1.73 m^2^ [24]. In NHANES, CVD history was confirmed if participants reported being diagnosed with heart failure, coronary heart disease (CHD), angina, heart attack, or stroke. Cancer history was collected through self-reported information during household interviews [25].

### 2.5. Outcomes

The NCHS has connected the 1999–2002 NHANES data to mortality records, allowing researchers to monitor the long-term health outcomes of participants. This linkage offers detailed information on both overall and cause-specific mortality, with follow-up extending through 2019. In this study, we gathered data on participants’ survival status and follow-up duration, classifying causes of death into three categories: all-cause mortality, cardiovascular mortality, and cancer-related mortality. Cardiovascular mortality was defined as death due to heart disease or cerebrovascular conditions. Comprehensive descriptions of the analytical methods used are available on the NCHS website [26].

### 2.6. Statistics

Because of the non-normal distribution, the natural logarithm (ln) of serum cystatin C, eGFRcr-cys, and DNAmCystatinC was applied in the analysis. Using the ln values of these three variables enabled the calculation of the exponential mean, allowing for the determination of geometric mean and standard error across different subgroups. Statistical analyses were conducted using two-tailed Student’s t-tests and one-way ANOVA. Sampling weights were applied to ensure that the findings were representative of the U.S. population [27]. We conducted multiple linear regression analyses with complex sampling to explore the relationship between serum cystatin C, eGFRcr-cys, and DNAmCystatinC. Model 1 was adjusted for age, sex, ethnicity, family poverty–income ratio, smoking status, alcohol consumption, and BMI. Model 2 included these variables and added adjustments for hypertension, hypercholesterolemia, diabetes, cardiovascular disease history, and cancer history. Model 3 further expanded on Model 2 by including an additional adjustment for CKD. Additionally, logistic regression for complex samples was conducted to assess the odds ratios (OR) of chronic diseases associated with a one-unit increase in the ln-transformed serum cystatin C, eGFRcr-cys, and DNAmCystatinC, adjusting for the covariates specified in Model 1. The hazard ratios (HR) for all-cause, cardiovascular, and cancer-related mortality were assessed for each one-unit increase in the ln-transformed values of serum cystatin C, eGFRcr-cys, and DNAmCystatin. A weighted Cox regression model was used for this analysis, adjusting for the covariates outlined in Models 2 and 3. Additionally, we assessed HR for all-cause mortality associated with a one-unit increase in different DNAmCystatinC and serum cystatin C subgroups divided by 50th percentile. Interaction effects were tested by incorporating cross-product terms into the regression models. All statistical analyses were conducted using SPSS version 20 (SPSS Inc., Chicago, IL, USA), with significance set at *p* < 0.05.

## 3. Results

The participants in the study had an average age of 68.72 years (SD = 8.71), with ages ranging from 50 to 85. During a median follow-up period of 192 months, a total of 981 deaths were documented, of which 318 were due to cardiovascular causes and 193 were cancer-related. Supplementary Table S1 provides detailed descriptive statistics (mean, SD, median, minimum, maximum, and interquartile range) for serum cystatin C, eGFRcr-cys, and DNAmCystatinC levels. Table 1 summarizes the demographic characteristics and biomarker levels of 1642 participants. Elevated serum cystatin C and DNAmCystatinC levels were associated with older adults (≥65 years), non-Hispanic white ethnicity, lower family poverty–income ratios, low alcohol consumption, hypertension, CVD, and a history of cancer. DNAmCystatinC levels were notably higher in men, participants with a BMI < 25, and non-smokers, whereas serum cystatin C levels were highest in individuals exposed to ETS. Supplementary Table S2 presents the OR and 95% CI for chronic diseases associated with a one-unit increase in ln-transformed serum cystatin C, eGFRcr-cys, and DNAmCystatinC, based on weighted logistic regression analyses. Higher ln-serum cystatin C was significantly associated with increased OR of hypertension and a history of CVD, but not with other chronic diseases. In contrast, higher levels of ln-eGFRcr-cys were associated with significantly lower OR of hypertension, diabetes, and CVD. Ln-DNAmCystatinC showed a significant positive association with CVD but did not demonstrate statistically significant relationships with other chronic conditions.

**Figure 2:**
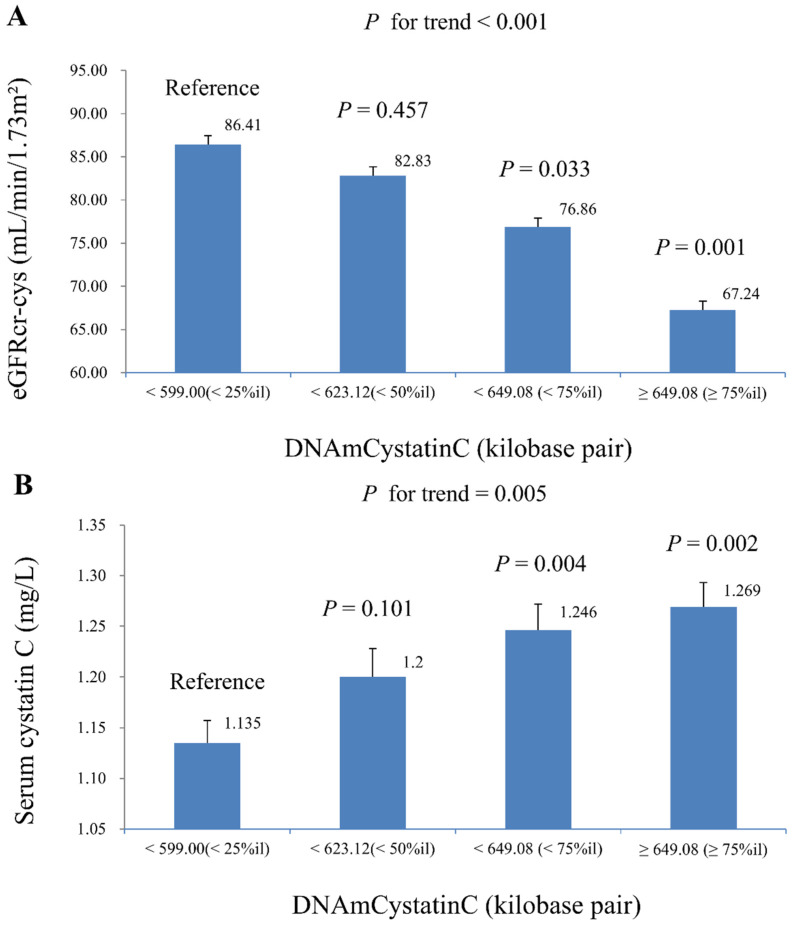
Geometric mean (S.E.) of eGFRcr-cys and serum cystatin C across quartiles of DNAmCystatinC in complex samples of multiple linear regression models, with results weighted for sampling strategy. (**A**) eGFRcr-cys (adjusted for model 2). (**B**) serum cystatin C (adjusted for model 3).

Table 2 summarizes the linear regression results examining the associations between ln-DNAmCystatinC, ln-serum cystatin C, and ln-eGFRcr-cys. DNAmCystatinC was significantly associated with both biomarkers across all models. In Model 2, a one-unit increase in ln-DNAmCystatinC was significantly associated with a decrease in ln-eGFRcr-cys (β =−1.123, *p* = 0.018). In Model 3, a one-unit increase in ln-DNAmCystatinC was significantly associated with an increase in ln-serum cystatin C (β = 0.773, *p* = 0.007). Figure 2 illustrates the geometric means of eGFRcr-cys and serum cystatin C across DNAmCystatinC quartiles, showing significant trends (*p* for trend < 0.001 and *p* = 0.005, respectively).

Supplementary Table S3 presents the linear regression coefficients for ln-serum cystatin C in response to a one-unit increase in ln-DNAmCystatinC within Model 3 across various subpopulations. Significant associations were observed in males, older adults (≥65 years), non-Hispanic whites, individuals with a BMI < 30 kg/m^2^, those consuming ≥12 alcoholic drinks per year, and all subgroups based on smoking status and kidney function. However, no significant interaction effects were found between these covariates and DNAmCystatinC on serum cystatin C levels. Supplementary Table S4 provides the linear regression coefficients for ln-eGFRcr-cys in response to a one-unit increase in ln-DNAmCystatinC within Model 2 across various subpopulations. Significant associations were noted in males, older adults, non-Hispanic whites, individuals with a BMI < 30 kg/m^2^, non-smokers, and those consuming ≥12 alcoholic drinks per year. An interaction effect between age and DNAmCystatinC on eGFRcr-cys was also detected.

**Figure 3:**
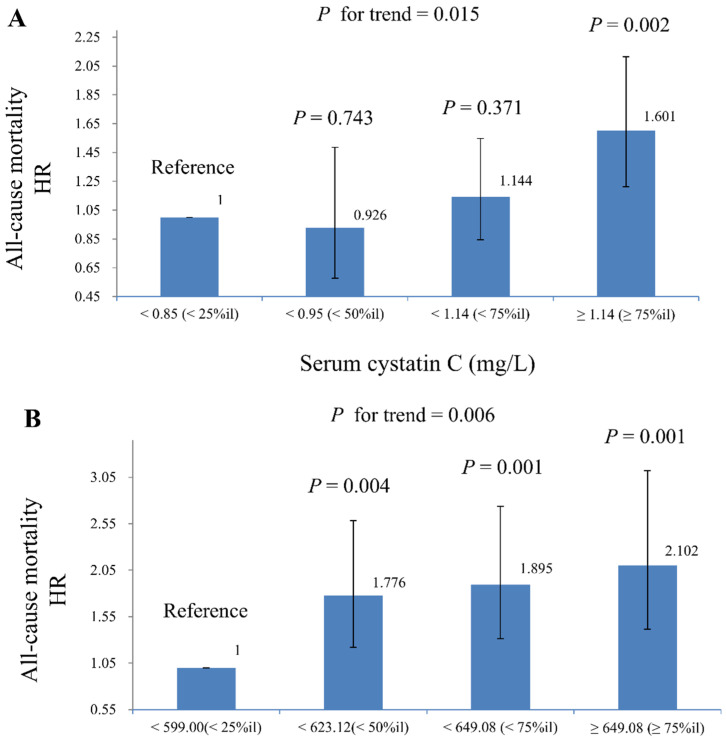
HR (95% CI) for all-cause mortality across quartiles of serum cystatin C and DNAmCystatinC in complex samples of Cox regression model (adjusted for model 3), with results weighted for sampling strategy. (**A**) Serum cystatin C. (**B**) DNAmCystatinC.

Table 3 presents the HR and 95% CI for all-cause, cardiovascular, and cancer-related mortality associated with serum cystatin C and DNAmCystatinC, based on weighted Cox regression models. An increase in serum cystatin C was significantly associated with higher risks of all-cause and cardiovascular mortality. In Model 3, each one-unit increase in ln-serum cystatin C corresponded to a 2.87-fold higher HR for all-cause mortality (95% CI: 1.938–4.26, *p* < 0.001) and a 3.04-fold higher HR for cardiovascular mortality (95% CI: 1.34–6.88, *p* = 0.010). No significant association was found with cancer-related mortality. Additionally, higher levels of DNAmCystatinC were positively linked to all-cause mortality. In Model 3, each one-unit increase in ln-DNAmCystatinC was associated with an HR of 135.86 (95% CI: 5.51–3349.69, *p* = 0.004) for all-cause mortality. In Model 2, each one-unit increase in eGFRcr-cys was significantly associated with a reduced HR of 0.48 for all-cause mortality (95% CI: 0.38–0.60, *p* < 0.001) and 0.57 for cardiovascular mortality (95% CI: 0.37–0.86, *p* = 0.009). Figure 3 displays the HR and 95% CI for all-cause mortality across quartiles of serum cystatin C and DNAmCystatinC, using a weighted Cox regression model. The analysis shows a significant increasing trend in mortality risk with higher quartiles of both biomarkers, with *p*-values for the trend of 0.015 for serum cystatin C and 0.006 for DNAmCystatinC. Notably, the HR for DNAmCystatinC from quartile 2 to quartile 4 were higher compared to serum cystatin C, and the *p*-values for the reference group (quartile 1) were more significant for DNAmCystatinC than for serum cystatin C.

Table 4 summarizes the HR and 95% CI for all-cause mortality in relation to a one-unit increase in ln-DNAmCystatinC across subgroups. Significant positive associations were found across both genders and all ethnicities. Younger age groups, individuals with lower BMI, active smokers, those exposed to ETS, those consuming ≥12 alcoholic drinks per year, and participants without CKD also showed significant positive associations. A significant interaction was observed between DNAmCystatinC and CKD on all-cause mortality (*p* for interaction = 0.002). Table 5 presents the HR and 95% CI for all-cause mortality across subgroups defined by serum cystatin C and DNAmCystatinC levels. The reference group consisted of individuals with serum cystatin C and DNAmCystatinC levels ≤ the 50th percentile. Participants with serum cystatin C and DNAmCystatinC levels > the 50th percentile exhibited the highest increase in mortality risk (HR = 1.85, 95% CI: 1.37–2.50). The trend of increasing mortality risk across these subgroups was statistically significant (*p* for trend <0.001). However, when including both Ln-cystatin C and Ln-DNAmCystatinC along with their interaction term in the model, the interaction term was not statistically significant (*p* = 0.078).

## 4. Discussion

In a nationally representative sample of U.S. adults, higher DNAmCystatinC levels were significantly associated with increased serum cystatin C and decreased eGFRcr-cys. Higher serum cystatin C levels were associated with increased all-cause and cardiovascular mortality, while elevated DNAmCystatinC showed a strong positive link with all-cause mortality. The effect of DNAmCystatinC on all-cause mortality was stronger in individuals without CKD, and a significant interaction between DNAmCystatinC and CKD emphasizes the need to consider renal function status when evaluating the impact of DNAmCystatinC on mortality. When cystatin C and DNAmCystatinC were each divided into quartiles, DNAmCystatinC exhibited higher HR and more significant *p*-values for all-cause mortality compared to serum cystatin C. This suggests that DNAmCystatinC may be a more effective predictor of all-cause mortality. In addition, higher levels of serum cystatin C and DNAmCystatinC above the 50th percentile are associated with the greatest risk of all-cause mortality. The non-significant interaction term indicates that their combined impact on all-cause mortality is likely additive rather than synergistic. Our study is the first to explore the relationships between DNAmCystatinC, serum cystatin C, eGFRcr-cys, and mortality in a representative U.S. population. While serum cystatin C is a well-established indicator of kidney health and mortality, this study extends existing research by investigating the independent and interactive effects of DNAmCystatinC, a methylation-based biomarker predicting cystatin C, on mortality. This finding suggests that DNAmCystatinC may be a stronger predictor of all-cause mortality. The additive effect of both biomarkers highlights their combined value in clinical practice, underscoring the importance of considering both biological aging and organ-specific health when assessing mortality risk. It highlights the importance of personalized approaches in epidemiological research and clinical practice.

Serum cystatin C has emerged as a reliable predictor of mortality across various clinical settings. A meta-analysis of cohort studies in elderly populations further confirmed its prognostic value, showing that elevated cystatin C levels were associated with a 74% higher risk of all-cause mortality and a doubled risk of cardiovascular mortality [28]. Notably, cystatin C also holds predictive value for mortality in individuals with normal renal function. Data from NHANES III demonstrated that higher cystatin C levels significantly increased risks of all-cause, cardiovascular, and non-cardiovascular mortality, independent of creatinine-based eGFR and other risk factors [11]. Additionally, cystatin C was strongly correlated with CHD prevalence, outperforming traditional markers like serum creatinine [12]. In this current study, our findings reveal that higher serum cystatin C levels are significantly associated with increased risks of all-cause and cardiovascular mortality, even after accounting for traditional risk factors including CKD. This finding is compatible with previous studies. Our study revealed that higher serum cystatin C levels were not associated with cancer mortality. In contrast, the large UK Biobank study, with over 241,000 participants, identified associations between higher cystatin C levels and mortality from specific cancers, including lung, blood, brain, esophageal, breast, and liver cancers [29]. This discrepancy could be explained by differences in sample size, statistical power, and population characteristics between the two studies.

With the aging population and advancements in technology, researchers have developed genome-wide DNA methylation profiles, known as epigenetic clocks, to estimate biological age. Early models, such as HorvathAge and HannumAge, were widely adopted [30]; however, more recent clocks demonstrate improved precision in predicting health outcomes. Advanced versions, including PhenoAge, GrimAge, and DNAm-based telomere length, offer more accurate assessments of mortality risk and other health-related indicators, marking significant progress in the field of biological aging [31]. GrimAge, in particular, integrates not only chronological age and sex but also seven plasma proteins—such as cystatin C and growth differentiation factor-15—and DNA methylation-based smoking pack-years to improve predictive power. These proteins were selected based on their associations with mortality [18,31]. A systematic review identified significant relationships between epigenetic clock acceleration and mortality, CVD, cancer, and diabetes [32]. These findings highlight the versatility of epigenetic clocks in capturing complex interactions between health, environment, and aging.

DNAmCystatinC is an epigenetic marker of cystatin C, derived from DNA methylation patterns associated with circulating cystatin C levels. It is integrated into advanced biological aging models, such as GrimAge and GrimAge2, to enhance the prediction of mortality and aging-related outcomes [18]. However, research on this biomarker and its associations with various health outcomes remains limited. In this current study, DNAmCystatinC exhibited a higher HR for all-cause mortality compared to serum cystatin C. This finding reinforces the hypothesis that this DNA methylation biomarker, which is incorporated into advanced biological aging models, serves as a more effective predictor of all-cause mortality in the study population [31]. While identifying an optimal cutoff value for DNAmCystatinC as a strong predictor of all-cause mortality is an important consideration, deriving such a threshold within the NHANES dataset is inherently complex due to its intricate survey design, weighted analysis requirements, and the nonlinear relationships between biomarkers and outcomes. Additionally, determining a universally applicable cutoff may be limited by population heterogeneity and variations in clinical contexts. Despite this limitation, our findings provide valuable insights into the association between DNAmCystatinC and mortality risk, highlighting its potential role in risk stratification and predictive modeling.

Several studies have demonstrated that epigenetic age acceleration—the gap between biological and chronological age based on DNA methylation—is associated with health outcomes [33]. For example, increased epigenetic age acceleration has been linked to higher cardiovascular mortality [33] and to specific cardiovascular events such as CHD and heart failure [34]. However, evidence regarding the relationship between epigenetic age acceleration and cancer remains mixed. While one study reported that blood-based epigenetic age could predict cancer incidence and mortality 3–5 years before onset [35], another found no significant association with overall breast cancer incidence [36]. In this study, DNAmCystatinC was not associated with cardiovascular or cancer mortality. As an epigenetic marker integrated into aging models like GrimAge, DNAmCystatinC may be more effective at predicting general aging-related outcomes rather than specific diseases. This lack of association could stem from its limited ability to capture the specific biological processes underlying cardiovascular and cancer mortality. Additionally, differences in findings may also be influenced by factors such as sample size, population characteristics, health status, and follow-up periods, which can impact the detection of associations between epigenetic markers and health outcomes.

Recent studies highlight the potential of DNA methylation biomarkers to predict mortality risk, complementing traditional clinical risk factors [37]. These biomarkers, when combined with traditional measures, can improve cardiovascular risk assessment [38]. In our current study, both higher levels of serum cystatin C and DNAmCystatinC were associated with an increased risk of all-cause mortality. The additive, rather than synergistic, effect between these biomarkers suggests that each independently contributes to mortality risk prediction without amplifying the other’s impact. The novelty of this finding lies in the independent and additive roles of DNAmCystatinC and serum cystatin C in predicting all-cause mortality. While prior research has examined DNA methylation biomarkers for mortality risk [31], our study uniquely shows that DNAmCystatinC provides insights beyond serum cystatin C levels, capturing additional biological information. This research positions DNAmCystatinC as a valuable tool for mortality risk assessment and highlights the need for incorporating both biomarkers into clinical evaluations, enhancing our understanding of the interplay between epigenetic factors and mortality.

CKD is associated with alterations in DNA methylation, which can influence disease progression and cardiovascular risk [39]. This aligns with our observation that DNAmCystatinC levels were elevated in CKD participants, as CKD disease-related changes in DNA methylation could contribute to the higher levels of DNAmCystatinC observed. Additionally, abnormal DNA methylation in CKD can drive inflammation and fibrosis, contributing to disease progression [40]. In our study, the effect of DNAmCystatinC on all-cause mortality was weaker among those with CKD, and the significant interaction between DNAmCystatinC and CKD was also reported. The result suggests that renal function status plays an important role in modifying the relationship between DNAmCystatinC and mortality risk. These epigenetic changes induced by CKD could potentially weaken the association between DNAmCystatinC and mortality in CKD patients, as CKD-related methylation alterations may affect the biological pathways captured by DNAmCystatinC [40]. Similarly, younger individuals and those with lower BMI demonstrated higher HR, which could suggest increased susceptibility to DNAmCystatinC-associated pathways in these populations or reduced interference from comorbid conditions that are more prevalent in older individuals and those with higher BMI. These findings highlight the complexity of DNAmCystatinC’s role in mortality risk prediction and emphasize the importance of considering renal function, age, and BMI as potential effect modifiers in future research.

The public health implications of this study are significant, particularly in enhancing early identification of individuals at risk for mortality. By demonstrating the strong association between DNAmCystatinC and all-cause mortality, especially in individuals with preserved kidney function, the findings suggest that DNAmCystatinC could serve as a valuable biomarker for risk stratification in clinical settings. The additive effect of cystatin C and DNAmCystatinC highlights their combined value in clinical practice. This has the potential to inform screening practices and improve patient management strategies, enabling healthcare providers to implement timely interventions for those identified as high-risk.

The study’s strengths lie in its robust design, leveraging a representative dataset from the NHANES, linked to comprehensive mortality data through the NCHS. This allows for a thorough exploration of the relationships between DNAmCystatinC, serum cystatin C, and eGFRcr-cys, which enhances the generalizability of the findings. Additionally, focusing on subpopulations allows for the identification of specific groups where DNAmCystatinC is particularly predictive of health outcomes, which can inform targeted interventions. However, limitations include the observational nature of the study, which precludes definitive causal conclusions. Furthermore, the smaller sample size available for DNAmCystatinC analyses further limits the robustness of findings. The reduced sample size may hinder precise estimations and the ability to draw firm conclusions, especially when stratifying by kidney function. Finally, while the study focuses on mortality risk, it does not explore the underlying mechanisms linking DNAmCystatinC with kidney function and health outcomes, necessitating further research to establish these relationships more clearly.

## 5. Conclusions

In conclusion, this study demonstrates significant associations between DNAmCystatinC, serum cystatin C, eGFRcr-cys, and all-cause mortality. Our findings highlight the stronger predictive value of DNAmCystatinC compared to serum cystatin C for all-cause mortality. The additive effect of both biomarkers suggests their potential utility as valuable indicators in clinical practice. The interaction between DNAmCystatinC and CKD suggests a complex relationship between kidney health and epigenetic aging. Incorporating DNAmCystatinC into routine assessments could enhance risk stratification and improve patient management, ultimately leading to better health outcomes. Future research should validate these findings and explore the broader implications of epigenetic markers in clinical and public health contexts.

## Figures and Tables

**Table 1:** Basic demographics of the sample subjects, including geometric means (geometric SE) of serum cystatin C, and DNAmCystatinC.

	N	Serum Cystatin C (mg/L)	*p*-Value	DNAmCystatinC (Kilobase Pair)	*p*-Value
Total	1642	1.008 (1.003)		623.576 (1.001)	
Sex			0.151		0.025
Men	836	1.018 (1.010)		625.531 (1.002)	
Women	806	0.997 (1.010)		621.554 (1.002)	
Age (in years)			<0.001		<0.001
50–65	583	0.919 (1.011)		594.870 (1.002)	
≥65	1059	1.060 (1.009)		639.966 (1.001)	
Ethnicity			<0.001		<0.001
Mexican-American	482	0.956 (1.014)		624.704 (1.002)	
Other Hispanic	88	0.993 (1.023)		617.930 (1.006)	
Non-Hispanic white	669	1.047 (1.010)		627.799 (1.002)	
Non-Hispanic black	352	1.019 (1.018)		616.392 (1.003)	
Other ethnicity	51	0.946 (1.037)		617.401 (1.009)	
Family poverty–income ratio			<0.001		<0.001
<1	294	1.046 (1.019)		628.626 (1.003)	
1–3	778	1.025 (1.011)		628.720 (1.002)	
>3	570	0.965 (1.011)		614.062 (1.002)	
Body mass index (kg/m^2^)			0.064		<0.001
<25	441	0.987 (1.014)		629.762 (1.003)	
25–30	653	1.003 (1.012)		623.989 (1.002)	
>30	548	1.030 (1.012)		618.154 (1.002)	
Smoking status			0.032		0.004
Non-smoker	1026	0.993 (1.009)		625.323 (1.002)	
ETS	334	1.039 (1.018)		623.468 (1.003)	
Current smoker	282	1.023 (1.018)		617.388 (1.003)	
Alcohol consumption (drinks/year)			0.012		0.001
<12	650	1.030 (1.015)		627.205 (1.002)	
≥12	992	0.993 (1.015)		621.209 (1.002)	
Hypertension			<0.001		<0.001
No	568	0.935 (1.009)		616.044 (1.002)	
Yes	1074	1.049 (1.010)		627.597 (1.002)	
Diabetes Mellitus			<0.001		0.345
No	1223	0.992 (1.008)		623.084 (1.002)	
Yes	419	1.053 (1.017)		625.014 (1.003)	
Hypercholesterolemia			0.892		0.454
No	1040	1.008 (1.010)		624.083 (1.001)	
Yes	602	1.006 (1.011)		622.700 (1.002)	
History of CVD			<0.001		<0.001
No	1292	0.974 (1.007)		620.713 (1.002)	
Yes	350	1.140 (1.019)		634.261 (1.003)	
History of cancer			0.036		<0.001
No	1396	1.001 (1.008)		621.751 (1.001)	
Yes	246	1.045 (1.016)		634.036 (1.004)	

Tested by two-tailed Student’s *t*-tests and one-way analysis of variance. Abbreviations: BMI: Body mass index; CKD: Chronic kidney disease; CVD: Cardiovascular disease; DNAmCystatinC: DNA methylation-predicted cystatin C; ETS: Environmental tobacco smoker.

**Table 2:** Linear regression coefficients (including standard errors) for the ln-eGFRcr-cys and ln-serum cystatin C in relation to a unit increase in ln-DNAmCystatinC, using multiple linear regression models weighted for the sampling strategy (Unweighted number/Population size = 1642/43,092,392).

	DNAmCystatinC (Kilobase Pair)	
	Adjusted β (SE)	*p*-Value
eGFRcr-cys (mL/min/1.73 m^2^)		
Model 1	−1.306 (0.534)	0.021
Model 2	−1.123 (0.449)	0.018
Serum cystatin C (mg/L)		
Model 1	1.134 (0.399)	0.008
Model 2	0.993 (0.334)	0.006
Model 3	0.773 (0.267)	0.007

Model 1 adjusted for age, sex, ethnicity, family poverty–income ratio, smoking, drinking, and BMI. Model 2 adjusted for model 1 plus hypertension, hypercholesterolemia, diabetes mellitus, a history of CVD, and a history of cancer. Model 3 adjusted for model 2 plus CKD. Abbreviations: BMI: Body mass index; CKD: Chronic kidney disease; DNAmCystatinC: DNA methylation-predicted cystatin C; eGFRcr-cys: Estimated glomerular filtration rate calculated using both creatinine and cystatin C.

**Table 3:** HR (95% CI) for all-cause, cardiovascular, and cancer-related mortality associated with a unit increase in ln-eGFRcr-cys, ln-serum cystatin, and ln-DNAmCystatinC. Results are derived from a weighted Cox regression model accounting for complex sampling design.

	Model 2		Model 3	
	HR (95% CI)	*p*-Value	HR (95% CI)	*p*-Value
Ln-eGFRcr-cys (mL/min/1.73 m^2^)				
All-cause mortality	0.48 (0.38–0.60)	<0.001		
Cardiovascular mortality *	0.57 (0.37–0.86)	0.009		
Cancer-related mortality	0.80 (0.36–1.75)	0.559		
Serum cystatin C (mg/L)				
All-cause mortality	3.04 (2.20–4.18)	<0.001	2.87 (1.938–4.26)	<0.001
Cardiovascular mortality *	2.53 (1.50–4.26)	0.001	3.04 (1.34–6.88)	0.010
Cancer-related mortality	1.60 (0.64–3.99)	0.305	1.01 (0.28–3.65)	0.991
DNAmCystatinC (kilobase pair)				
All-cause mortality	238.82 (11.02–5174.95)	0.001	135.86 (5.51–3349.69)	0.004
Cardiovascular mortality *	40.70 (0.08–21,934.33)	0.238	28.53 (0.05–14,967.83)	0.283
Cancer-related mortality	25.69 (0.03–19,764.14)	0.326	15.26 (0.02–10,671.01)	0.402

eGFRcr-cys and serum cystatin C: Unweighted number/Population size = 1642/31,597,803. DNAmCystatinC: Unweighted number/Population size = 1642/43,092,392. Model 2 adjusted for age, sex, ethnicity, family poverty–income ratio, smoking, drinking, BMI, hypertension, hypercholesterolemia, diabetes mellitus, a history of CVD, and a history of cancer. Model 3 adjusted for model 2 plus CKD. * Cardiovascular mortality: Death from heart or cerebrovascular disease. Abbreviations: CKD: Chronic kidney disease; DNAmCystatinC: DNA methylation-predicted cystatin C; eGFRcr-cys: Estimated glomerular filtration rate calculated using both creatinine and cystatin C; HR: Hazard ratios.

**Table 4:** HR (95% CI) for all-cause mortality associated with a 10-unit increase in DNAmCystatinC across subpopulations. Results are based on a weighted Cox regression model, accounting for the complex sampling design.

DNAmCystatinC (Kilobase Pair)	Unweighted Number/ Population Size	All-Cause Mortality		
		HR (95% CI)	*p*-Value	*p* for Interaction
Gender				0.661
Men	836/19,240,990	1.07 (1.01–1.15)	0.033	
Women	806/23,851,402	2.11 (1.04–4.26)	0.018	
Age, years				0.681
50–64	583/16,069,197	1.84 (1.10–3.09)	0.010	
≥65	1.059/27,023,195	1.58 (0.99–2.54)	0.059	
Ethnicity				0.644
Non-Hispanic white	669/34,366,802	1.80 (1.01–3.20)	0.047	
Other	973/8,725,590	1.07 (1.02–1.12)	0.008	
BMI (kg/m^2^)				0.303
<30	1094/29,164,479	1.76 (1.06–2.91)	0.010	
≥30	548/13,927,913	1.26 (0.99–1.61)	0.075	
Smoking status				0.848
Active smoker and ETS	616/15,995,657	1.62 (1.02–2.58)	0.043	
Non-smoker	1026/27,096,734	1.45 (0.99–1.90)	0.081	
Alcohol consumption (drinks/year)				0.056
<12	650/16,198,402	1.04 (0.65–1.71)	0.785	
≥12	992/26,893,989	1.86 (1.27–2.73)	<0.001	
CKD				0.002
No	1374/36,086,885	1.77 (1.19–2.64)	<0.001	
Yes	268/7,005,507	0.96 (0.71–1.29)	0.530	

Adjusted for model 3. Abbreviations: CKD: Chronic kidney disease; DNAmCystatinC: DNA methylation-predicted cystatin C; HR: Hazard ratios.

**Table 5:** HR (95% CI) for all-cause mortality in different serum cystatin C and DNAmCystatinC subgroups in complex sample of Cox regression models, with results weighted for sampling strategy.

		All-Cause Mortality				
Serum Cystatin C (mg/L)	DNAmCystatinC (Kilobase Pair)	Unweighted Number/Population Size	HR (95% CI)	*p*-Value	*p* for Trend	*p* for Interaction
≤0.950 (50%ile)	≤623.124 (50%ile)	511/14,524,136	1	Reference	<0.001	0.078
	>623.124 (50%ile)	274/5,165,348	1.31 (0.92–1.86)	0.219		
>0.950 (50%ile)	≤623.124 (50%ile)	310/9,500,488	1.64 (1.22–2.20)	0.002		
	>623.124 (50%ile)	547/13,092,392	1.85 (1.37–2.50)	<0.001		

Adjusted for model 3. Abbreviations: DNAmCystatinC: DNA methylation-predicted cystatin C; HR: Hazard ratios.

## Data Availability

The datasets analyzed during the current study are available at the NHANES website (https://www.cdc.gov/nchs/nhanes/index.htm (accessed on 27 October 2024)).

## Supplementary Materials

The following supporting information can be downloaded at: https://www.mdpi.com/article/10.3390/life15010013/s1, Table S1: The levels of serum cystatin C, eGFRcr-cys, and DNAmCystatinC; Table S2: OR (95% confidence interval [CI]) of chronic diseases with one unit increase in ln-serum cystatin C, and ln-DNAmCystatinC, and ln-eGFRcr-cys in complex samples of logistic regression analysis, with results weighted for sampling strategy; Table S3: Linear regression coefficients (SE) of ln-serum cystatin C with a unit increase in ln-DNAmCystatinC in multiple linear regression models in subpopulation, with results weighted for sampling strategy; Table S4: Linear regression coefficients (SE) of ln-eGFRcr-cys with a unit increase in ln-DNAmCystatinC in multiple linear regression models in subpopulation, with results weighted for sampling strategy.

## Abbreviations

BMI	Body mass index
CHD	Coronary heart disease
CKD	Chronic kidney disease
CVD	Cardiovascular disease
DNAmCystatinC	DNA methylation-predicted cystatin C
eGFR	Estimated glomerular filtration rate
eGFRcr-cys	Estimated glomerular filtration rate calculated using both creatinine and cystatin C
ETS	Exposed to secondhand smoke
HR	Hazard ratios
Ln	Natural logarithm
LOD	Limit of detection
NCHS	National Center for Health Statistics
NHANES	National Health and Nutrition Examination Survey
OR	Odds ratios
