# Supplementary material for: Associations of Serum Cystatin C, DNAm Cystatin C, Renal Function, and Mortality in U.S. Adults
Source: Life (Basel). 2024 Dec 27;15(1):13. doi: 10.3390/life15010013 (PMC11767232; doi:10.3390/life15010013)
Supplement: Supplementary file 1 [file life-15-00013-s001.zip › life-3374602-supplementary.pdf]

Supplementary material

Supplemental Table S1. The levels of serum cystatin C, eGFRcr-cys, and DNAmCystatinC.

	Serum cystatin C (mg/L)	eGFRcr-cys (mL/min/1.73m²)	DNAmCystatinC (kilobase pair)
Mean	1.07	82.41	624.62
Standard deviation	0.53	21.92	36.25
Median	0.95	85.25	623.12
Minimum	0.43	4.71	516.56
Maximum	7.56	130.29	789.20
Interquartile range	0.29	29.37	50.09

Abbreviations: DNAmCystatinC: DNA methylation predicted cystatin C; eGFRcr-cys: Estimated glomerular filtration rate calculated using both creatinine and cystatin C.

Supplemental Table S2. OR (95% confidence interval [CI]) of chronic diseases with one unit increase in ln-serum cystatin C, and ln-DNAmCystatinC, and ln-eGFRcr-cys in complex samples of logistic regression analysis, with results weighted for sampling strategy.

	Ln-serum cystatin C (mg/L)		Ln-DNAmCystatinC (kilobase pair)		Ln-eGFRcr-cys (mL/min/1.73m²)	
	OR (95% CI)	P value	OR (95% CI)	P value	OR (95% CI)	P value
Hypertension	2.448 (1.330—4.509)	0.006	1.461 (0.013—170.411)	0.872	0.460 (0.283—0.746)	0.003
Diabetes Mellitus	1.890 (0.901—3.967)	0.089	1.264 (0.020—80.987)	0.909	0.535 (0.306—0.935)	0.029
Hypercholesterolemia	0.715 (0.406—1.258)	0.234	0.454 (0.012—15.533)	0.662	1.175 (0.741—1.865)	0.480
History of CVD	4.832 (2.781—8.398)	<0.001	764.206 (3.699—157900.753)	0.016	0.291 (0.193—0.440)	<0.001
History of cancer	1.319 (0.586—2.967)	0.490	166.468 (0.184—150906.059)	0.135	0.799 (0.454—1.404)	0.422
Chronic kidney disease	3.48×10^9 (4.69×10^7—2.58×10^{11})	<0.001	282.523 (0.870—91788.514)	0.055		

Adjusted for Model 1: Age, sex, ethnicity, family poverty income ratio, smoking, drinking, and BMI.

Abbreviations: BMI: Body mass index; CVD: Cardiovascular diseases; DNAmCystatinC: DNA methylation predicted cystatin C; Estimated

glomerular filtration rate calculated using both creatinine and cystatin C; Odds ratios: OR.

Supplemental Table S3. Linear regression coefficients (SE) of ln-serum cystatin C with a unit increase in ln-DNAmCystatinC in multiple linear regression models in subpopulation, with results weighted for sampling strategy.

	Unweighted no./ Population size	Ln-serum cystatin C (mg/L)		
		Adjusted β (SE)	*P* value	*P* for interaction
Gender				0.822
Men	836/19,240,990	0.726 (0.272)	0.012	
Women	806/23,851,402	0.738 (0.484)	0.139	
Age, years				0.639
50-64	583/16,069,197	0.879 (0.475)	0.075	
≥ 65	1.059/27,023,195	0.722 (0.204)	0.001	
Ethnicity				0.391
Non-Hispanic white	669/34,366,802	1.052 (0.342)	0.005	
Other	973/8,725,590	0.167 (0.238)	0.490	
BMI (kg/m^2)				0.516
< 30	1,094/29,164,479	0.930 (0.354)	0.014	
≥ 30	548/13,927,913	0.299 (0.256)	0.253	
Smoking status				0.159
Active smoker and ETS	616/15,995,657	1.264 (0.559)	0.031	
Non-smoker	1,026/27,096,734	0.414 (0.184)	0.032	
Alcohol consumption (drinks/year)				0.380
< 12	650/16,198,402	0.346 (0.232)	0.147	

≥ 12	992/26,893,989	1.111 (0.291)	0.001	
CKD				0.102
No	1,374/36,086,885	0.482 (0.149)	0.003	
Yes	268/7,005,507	2.409 (1.009)	0.025	

Adjusted for model 3.

Abbreviations: BMI: Body mass index; CKD: Chronic kidney disease; DNAmCystatinC: DNA methylation predicted cystatin C; ETS: Environmental tobacco smoker.

Supplemental Table S4. Linear regression coefficients (SE) of ln-eGFRcr-cys with a unit increase in ln-DNAmCystatinC in multiple linear regression models in subpopulation, with results weighted for sampling strategy.

	Unweighted no./ Population size	Ln-eGFRcr-cys (mL/min/1.73m²)		
		Adjusted β (SE)	*P* value	*P* for interaction
Gender				0.653
Men	836/19,240,990	-0.992 (0.395)	0.018	
Women	806/23,851,402	-1.407 (0.997)	0.169	
Age, years				0.001
50-64	583/16,069,197	-0.787 (0.901)	0.390	
≥ 65	1.059/27,023,195	-1.533 (0.336)	<0.001	
Ethnicity				0.421
Non-Hispanic white	669/34,366,802	-1.695 (0.746)	0.032	
Other	973/8,725,590	-0.505 (0.412)	0.230	
BMI (kg/m^2)				0.402
< 30	1,094/29,164,479	-1.479 (0.705)	0.045	
≥ 30	548/13,927,913	-0.868 (0.602)	0.160	
Smoking status				0.909
Active smoker and ETS	616/15,995,657	-2.343 (1.336)	0.090	
Non-smoker	1,026/27,096,734	-0.606 (0.178)	0.002	
Alcohol consumption (drinks/year)				0.437
< 12	650/16,198,402	-0.811 (0.439)	0.075	

≥ 12	992/26,893,989	-1.720 (0.735)	0.026

Adjusted for model 2.

Abbreviations: BMI: Body mass index; DNAmCystatinC: DNA methylation predicted cystatin C; eGFRcr-cys: Estimated glomerular filtration rate calculated using both creatinine and cystatin C; ETS: Environmental tobacco smoker.
